# Single nanowire-based UV photodetectors for fast switching

**DOI:** 10.1186/1556-276X-6-348

**Published:** 2011-04-19

**Authors:** Kamran ul Hasan, N H Alvi, Jun Lu, O Nur, Magnus Willander

**Affiliations:** 1 Department of Science and Technology (ITN) Linköping University, Campus Norrköping, SE-601 74 Norrköping, Sweden; 2 Thin Film Physics, IFM, Linköping University, Linköping, 581 83, Sweden

## Abstract

Relatively long (30 µm) high quality ZnO nanowires (NWs) were grown by the vapor-liquid-solid (VLS) technique. Schottky diodes of single NW were fabricated by putting single ZnO NW across Au and Pt electrodes. A device with ohmic contacts at both the sides was also fabricated for comparison. The current-voltage (*I*-*V*) measurements for the Schottky diode show clear rectifying behavior and no reverse breakdown was seen down to -5 V. High current was observed in the forward bias and the device was found to be stable up to 12 V applied bias. The Schottky barrier device shows more sensitivity, lower dark current, and much faster switching under pulsed UV illumination. Desorption and re-adsorption of much smaller number of oxygen ions at the Schottky junction effectively alters the barrier height resulting in a faster response even for very long NWs. The NW was treated with oxygen plasma to improve the switching. The photodetector shows high stability, reversibility, and sensitivity to UV light. The results imply that single ZnO NW Schottky diode is a promising candidate for fabricating UV photodetectors.

## Introduction

Zinc oxide (ZnO) is a unique material with semiconducting and piezoelectric dual properties. It is turning out to be a very important material due to its wide variety of potential applications in everyday life like sunscreens, miniaturized lasers, light sources, sensors, piezoelectric elements for power nano-generators, transparent electrodes [1] etc. ZnO has many advantages over other wide bangap semiconductors like direct band gap of 3.37 eV, large excitons binding energy of 60 meV, high thermal/chemical stabilities, and the option of wet chemical etching etc. [1,2]. This has led to the demonstration of ZnO as an alternative material to the nitride semiconductors.

ZnO has a rich family of nanostructures such as nanowires, nano belts, nano particles, nano tips, and nanotubes [1,3]. ZnO nanowires (NWs) have attracted significant attention due to their large surface area, good crystal quality, and unique photonic properties. One-dimensional nanocrystal, for instance, a NW can serve as a sample for studying the low-dimensional phenomena and is potentially a building block for the complex nanodevices.

P-type doping of ZnO is still a problem that diminishes the prospects of a ZnO *p*-*n *homojunction device [4]. On the other hand, ZnO is naturally *n*-doped and does not need external dopants. A Schottky diode seems to be a very feasible device from ZnO. A Schottky barrier diode exhibits faster switching and lower turn-on voltages as compared to a *p*-*n *junction diode and there is some optical loss in the *p*-region of a *p*-*n *diode. That makes it a very useful for electronic and optoelectronic application.

In the past few years, there has been an increased interest in one-dimensional NW based UV sensors and these demonstrated potential applications as next-generation of UV sensors [5-8]. However, there are relatively much less reports on comparative study of photosensitivity dependence on the type of metal semiconductor junction. This article reports our UV response measurements of a Schottky-junction diode made of a single ZnO NW in comparison with a ZnO NW with ohmic contacts on both the sides. Very long NWs (approx. 30 µm) were used in this study that show very fast response on full length device (due to the reduced dimensionality of the active area at the Schottky junction) and potentially allows fabrication of several diodes on a single NW.

## Experimental

**Figure 1:**
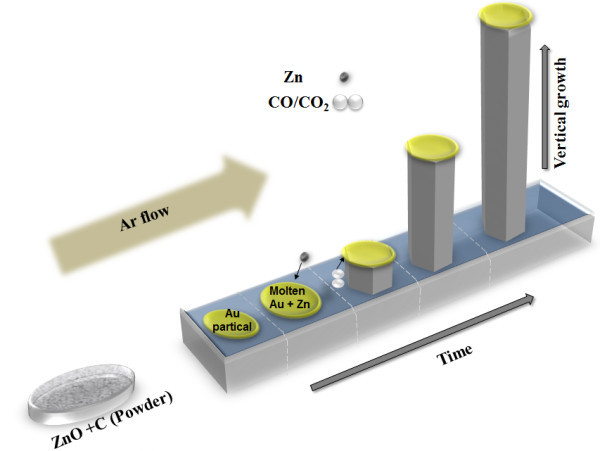
**Schematic of the VLS growth process**.

Relatively long (30 to 40 µm) crystalline ZnO NWs with a lateral diameter of approx. 100 nm were grown by high temperature (approx. 900°C) vapor-liquid-solid (VLS) technique. For the growth of ZnO nanowires, a thin film of pure Au (99.9%) was used as a catalyst and was deposited on the Si substrate in a high vacuum metallization chamber. The thin gold film melts into small gold droplets at elevated temperature, which act as growth sites for ZnO nanowires. The source material was prepared by mixing graphite (99.9%) with ZnO (99.9%) powder with ratio of 1:1. The source material was placed into a ceramic boat and the substrate was placed 3 to 4 cm away in the downstream and the growth face was downward to the source material. Zn, CO, and CO_2 _gases are produced from the reaction of ZnO and graphite powder at 900°C. Zn atoms adsorb on the Au droplet surface due to higher sticking coefficient of Zn on liquid versus solid. CO/CO_2 _molecules are transported to the liquid-solid interface and bulk diffusion of Zn takes place through Au droplet [9]. Zn islands oxidize to ZnO due to the presence of CO/CO_2 _mixture. The argon gas was used as a carrier gas with flow of 50 to 80 sccm (standard cubic centimeters per minute). The growth temperature was approx. 900°C. The growth time was about 40 min. The schematic of the process is shown in Figure 1.

**Figure 2:**
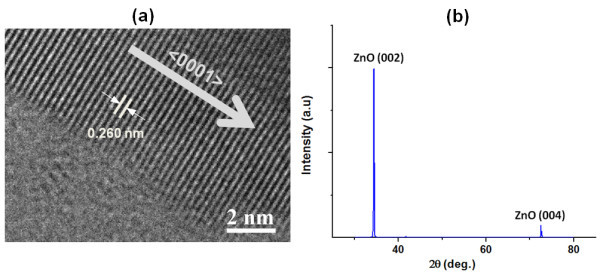
**Structural characterization of the ZnO nanowires**. (a) HRTEM image of the edge of an as-synthesized ZnO nanowire. The spacing of 0.26 nm between adjacent lattice planes corresponds to (002) lattice planes of ZnO and <0001> growth direction is also shown; (b) XRD spectrum of the ZnO nanowires.

The samples were annealed at 600°C in ambient argon to improve the crystal quality and minimize the defects. High-resolution transmission electron microscopy (HRTEM) image (Figure 2a) indicates the good monocrystalline quality structure of the ZnO NW. Lattice spacing is approximately 0.26 nm between the two adjacent (002) lattice planes and it confirms the <0001> growth direction [10]. Furthermore, the X-ray diffraction (XRD) pattern of the ZnO nanowires is shown in Figure 2b. The strong (002) peak and weak (004) peak reconfirm that the ZnO nanowires preferentially grow along the *c*-axis <0001> direction. Higher intensity and narrow spectral width of the (002) peak affirms that the grown ZnO has high-purity wurtzite hexagonal phase [11].

**Figure 3:**
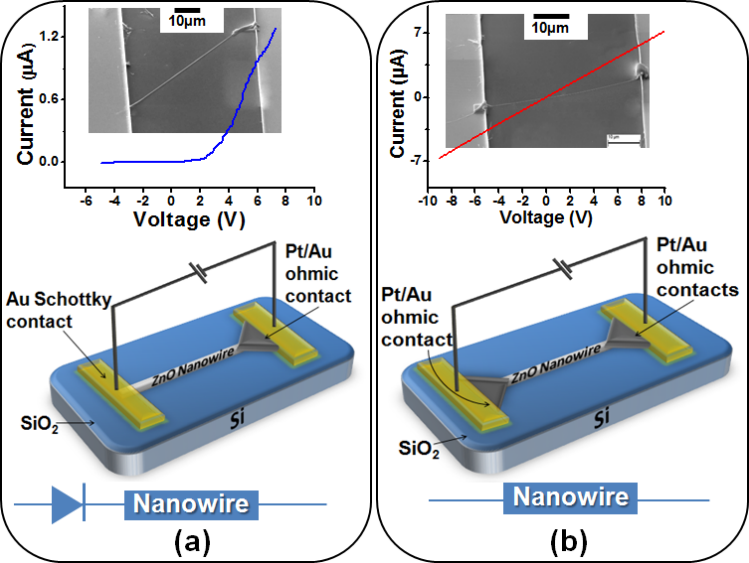
**Two types of devices fabricated for comparison**. (a) Electric model, schematic and SEM of the fabricated Schottky diode, I-V showing good rectifying behavior; and (b) Electric model, schematic and SEM of the device with ohmic contact on both sides, I-V show clear ohmic behavior.

NWs were then ultrasonically dispersed in ethanol and were placed onto a gold patterned insulated SiO_2 _substrate. After drying out the suspension, gold was re-evaporated onto one side of some selected ZnO NWs by lithography and lift-off process in order to form a stable Schottky contact. Focused ion beam (FIB) is used to deposit Ga induced Pt on the contact between the NW and the gold electrodes for eliminating the Schottky barriers and form high quality ohmic contacts [12]. Some samples were prepared by making ohmic contacts to both the sides. The scanning electron microscopic (SEM) images and the schematics of the device are shown in the Figure 3.

## Results and discussion

In Figure 2b, the *I*-*V *characteristics show a linear behavior between the two Pt ohmic contacts at the room temperature. This verifies that both the Pt electrodes show a good ohmic behavior. The *I*-*V *characteristics of our ZnO NW Schottky diode shown in Figure 3a demonstrate a good rectifying behavior.

Threshold voltage was also observed to be shifted from 2 to 1.2 V as temperature rise from 80 to 340 K. Electron transport through the Schottky barrier is described by thermionic emission as well as by small tunneling current. The thermionic current produces the rectifying *I*-*V *curve and dominates electron transport. The forward bias *I*-*V *characteristics in the Schottky-junction diode were analyzed using the thermionic emission model given by [13,14];

where *η *is the ideality factor, *k *the Boltzmann constant, *T *the temperature, *R*_s _is the series resistance, and *I*_SAT _is the reverse saturation current. The ideality factor values were found to be >3, which indicates that some non-thermionic processes also contribute to the conduction [13,15]. The barrier height is calculated from the relation:

where *A *is the area of the diode, φ_b_the Schottky barrier height (SBH) of the junction, and A* the Richardson constant, which is 32 A cm^-2 ^K^-2 ^for ZnO [15]. SBH was calculated to be 0.48 eV with an ideality factor of 3.1. These unusual electrical characteristics of our single ZnO NW Schottky diode can be explained by a thermionic field emission and an enhancement of the tunneling effects due to both the naturally high carrier concentration of the ZnO NW itself and the nanoscale junction size of the NW Schottky diodes [13].

**Figure 4:**
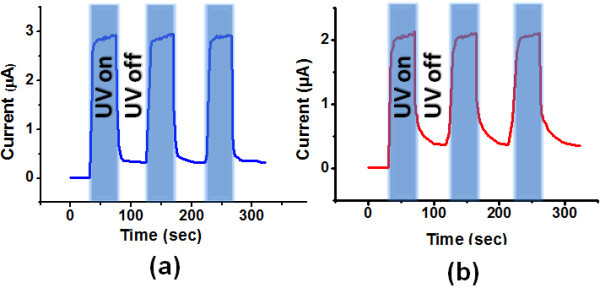
**Photoresponse of a single ZnO nanowire under pulsed illumination by a 365 nm wavelength UV light with (a) Schottky contact on one side, and (b) ohmic contacts on both sides**.

Photoconductive response is a key figure of merit for a photodetector. Response of both the ohmic and Schottky devices was measured using a 365-nm UV source at a bias of 0.5 V (Figure 4). The real time ON/OFF switching was measured by applying a UV pulse with an intensity of 1.5 mW/cm^2^. The measured photocurrent shows a rapidly rise and fall upon exposure to UV light for the Schottky detector and the current decreases down to 35 nA, which is quite close to the initial value under dark. Photocurrent pulse shows good stability and reversibility. Whereas, the recovery time is much higher for the ohmic detector and the value of the dark current is also relatively higher. Thus, the Schottky diode shows much faster switching under pulsed UV illumination as compared to the device with ohmic contacts on both the sides.

Under dark condition, oxygen molecules are adsorbed on the NW surface and capture free electrons from the *n*-type ZnO, making negatively charged O_2 _ions at the surface. This creates a low conductivity depletion layer near the NW surface:

When the UV exposure is made, electron-hole pairs are photo-generated and holes are trapped at the surface by the oxygen ions via surface electron-hole recombination:

Unpaired electrons are left behind which add to the photocurrent [5,6]. Thus, the NWs are very suitable for obtaining higher sensitivity of the devices due to an enhanced surface to volume ratio. Schottky barrier demonstrates hole-trapping in the reversed bias junction that reduces the depletion region and assists tunneling of additional electrons [16].

When the UV illumination is switched on or off, the oxygen is desorbed or readsorbed in the interfacial region in the premises of the metal contact in Schottky diodes and it reduces the Schottky barrier height, whereas for the device with ohmic contacts on the both sides, it happens throughout the NW surface. This explains the better sensitivity and faster switching of the photocurrent in the Schottky barrier devices as compared to the device with ohmic contact on both sides. This can be useful for carrying out single photon detection [5] as the adsorption and desorption of small number of oxygen ions at the junction area can effectively alter the barrier height. Usually it was considered advantageous to use short length NW for faster switching but with this Schottky barrier approach, even the longer NWs (approx. 30 µm in our case) are equally responsive. This allows for the possibility of processing multichannel NW devices with conventional photolithography as on most of the previous occasions [5,13,17,18] e-beam lithography is compulsory due to the very small NW lengths.

**Figure 5:**
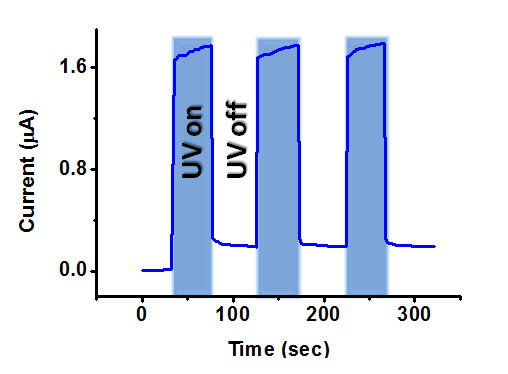
**Photoresponse of a single ZnO nanowire Schottky diode under pulsed illumination after oxygen plasma treatment**.

The NW was then treated with oxygen plasma under an oxygen flow rate of 100 sccm, chamber pressure of 150 mTorr for 1 min. Photocurrent was observed to decrease after the oxygen plasma treatment but photocurrent rise and fall time under UV exposure is further improved significantly as compared to the untreated ZnO NW, as shown in Figure 5.

Oxygen vacancies act as electron donors inside ZnO. Oxygen plasma treatment causes oxygen ions to diffuse into the ZnO NW to fill the oxygen vacancies. This results in the reduction of the total photocurrent. Whereas, surface defects and charged species for trapping and scattering the carriers increase after the oxygen plasma treatment thus this surface modification works in favor of faster switching.

## Conclusion

In summary, Schottky diodes of very long (approx. 30 µm) single NW were fabricated by putting single ZnO NW across Au and Pt (Ga induced) electrodes. A device with ohmic contacts to both the sides was also fabricated for comparison. UV photoconductive response of both the ohmic and Schottky devices was measured. The Schottky barrier device shows more sensitivity, lower dark current, and much faster switching under pulsed UV illumination. Desorption and re-adsorption of much smaller number of oxygen ions at the Schottky junction effectively alter the barrier height resulting in a faster response even for very long NWs, thus making possible the processing of the device by conventional techniques. The oxygen plasma treatment further enhances the switching. The photodetector show high stability, reversibility, and sensitivity to the UV light. Thus, a complete recipe for a UV photodetector capable of fast switching is concluded out of the present research.

## Abbreviations

FIB: focused ion beam; HRTEM: high-resolution transmission electron microscopy; NWs: nanowires; SBH: Schottky barrier height; SEM: scanning electron microscopic; VLS: vapor-liquid-solid; XRD: X-ray diffraction; ZnO: zinc oxide.

## Competing interests

The authors declare that they have no competing interests.

## Authors' contributions

All authors contributed equally, read and approved the final manuscript.
